# Rashba Spin Splitting in HgCdTe Quantum Wells with Inverted and Normal Band Structures

**DOI:** 10.3390/nano12071238

**Published:** 2022-04-06

**Authors:** Svetlana V. Gudina, Vladimir N. Neverov, Mikhail R. Popov, Konstantin V. Turutkin, Sergey M. Podgornykh, Nina G. Shelushinina, Mikhail V. Yakunin, Nikolay N. Mikhailov, Sergey A. Dvoretsky

**Affiliations:** 1 M.N. Mikheev Institute of Metal Physics of Ural Branch of Russian Academy of Sciences, 620108 Yekaterinburg, Russia; neverov@imp.uran.ru (V.N.N.); popov_mr@imp.uran.ru (M.R.P.); turutkin_kv@imp.uran.ru (K.V.T.); sp@imp.uran.ru (S.M.P.); shel@imp.uran.ru (N.G.S.); yakunin@imp.uran.ru (M.V.Y.); 2 A.V. Rzhanov Institute of Semiconductor Physics of Siberian Branch of Russian Academy of Sciences, 630090 Novosibirsk, Russia; mikhailov@isp.nsc.ru (N.N.M.); dvor@isp.nsc.ru (S.A.D.)

**Keywords:** Rashba spin splitting, HgTe, quantum wells, Shubnikov-de-Haas oscillations

## Abstract

In quantum wells (QWs) formed in HgCdTe/CdHgTe heterosystems with a variable composition of Cd(Hg), Shubnikov-de-Haas (SdH) oscillations are investigated to characterize the Rashba-type spin-orbit coupling in QWs with both a normal and inverted band structure. Several methods of extracting the Rashba spin-splitting at zero magnetic field and their magnetic field dependences from the beatings of SdH oscillations are used for greater reliability. The large and similar Rashba splitting (25–27 meV) is found for different kinds of spectrum, explained by a significant fraction of the p-type wave functions, in both the E1 subband of the sample with a normal spectrum and the H1 subband for the sample with an inverted one.

## 1. Introduction

The main spin-dependent interaction in non-magnetic semiconductors is the spin-orbit interaction. Depending on the crystal symmetry, as well as on the structural properties of semiconductor heterostructures, the spin-orbit coupling takes different functional forms, providing a good selection of systems with different effective spin-orbit Hamiltonians. spin-orbit interaction leads to spin-splitting of energy levels in zero magnetic field for the states with sufficiently lowered symmetry, in both bulk (3D) systems and two-dimensional (2D) quantum wells.

The spin-orbit splitting of electron states in quantum wells (QWs) is usually discussed in terms of the Rashba spin-orbit coupling arising from the structure inversion asymmetry (SIA) of the 2D object [1,2,3,4], the Dresselhaus spin-orbit coupling arising due to the bulk inversion asymmetry in the noncentrosymmetric crystals and causing cubic *k* terms in the spectrum (the BIA contribution) [5,6,7], and the contribution from the inversion asymmetry of the interface (IIA) [8,9,10], as well as their combinations.

The idea of manipulating the charge carrier spin remains a hot topic in condensed matter physics; thus, the search for materials with prominent spin-dependent properties and the possibility of their improvement are of interest. The removal of spin degeneracy in the absence of a magnetic field is a topic of constant interest in the study of heterostructures based on narrow-gap and gapless semiconductors. The Rashba effect was studied in II–VI HgTe quantum wells, where the typical values of the Rashba splitting energy range from 17 meV [11] to 30 meV [12,13,14,15,16,17], which is noticeably larger than for the narrow-gap III–V systems (3–5 meV) (see, for example, [18] and references therein). In order to realize the spintronic device that operates at room temperature, the Rashba spin-splitting energy, Δ*_R_*, higher than thermal energy of 26 meV is desirable [19]. These are exactly the values that were obtained in structures based on the mercury telluride with an inverted band structure.

HgCdTe solid solutions contain heavy elements; therefore, they are semiconductors with a strong spin-orbit interaction [20,21]. CdTe has a positive band gap, so its band structure is similar to that of other conventional semiconductors. The conduction band states have *s*—symmetry (Γ6), and the states of the valence band have *p*—symmetry (Γ8). In the gapless semiconductor HgTe, due to relativistic effects [20], the Γ8 band normally forms the conduction band located above the Γ6 band, thus constituting an inverted band structure. An increase in the Cd content in the Hg_1−*x*_Cd*_x_*Te solid solution causes a transition from an inverted to a normal band structure at *x* = 0.16 [21].

In the narrow HgTe quantum well within the Cd(Hg)Te/HgTe/Cd(Hg)Te heterostructure, two-dimensional electronic states have a normal band order (CdTe-like), but the sequence of the bands becomes inverted (HgTe-like) for *d* > *d_c_*, where *d_c_* is the critical width [22]. For HgTe/Cd_0.7_Hg_0.3_Te QWs grown on CdTe buffer, *d_c_* ≈ 6.5 nm. The critical width depends on the crystallographic orientation, buffer material and, especially, on the Cd content in Cd(Hg)Te solid solution in both the quantum well and the barriers. This provides an additional way of manipulating the properties of heterostructures with a HgTe (or CdHgTe) QW.

It is the inverted nature of HgTe band structure, i.e., the *p*-like character of energy states with the *z*-projection of total angular momentum J_z_ = ±3/2, that is responsible for the large Rashba spin-splitting. There are a few investigations of Rashba spin-splitting in HgTe QW with an inverted band order [11,12,13,14,23] that demonstrate a large spin-splitting. The triangle QWs in inversion layers on HgCdTe with normal [24] and inverted [15] band structures grown by the liquid-phase epitaxy technique were studied, and the latter demonstrated the record spin-splitting of 34 meV.

In Reference [25], the spectra of the cyclotron resonance in the classical and quantizing magnetic fields in asymmetric HgCdTe/CdHgTe heterostructures with selective doping in barriers were investigated. Self-consistent calculations of energy spectra (at *B* = 0) and Landau levels in a standard 8-band Kane model in Hartree approximation were performed. In low fields, a strong splitting of the cyclotron resonance line (10%), associated with the Rashba effect, was found in both a sample with an inverted band structure and a normal one. The evolution of absorption lines with a magnetic field of up to 34 T was traced when magnetic quantization prevailed over the Rashba splitting.

This paper presents the results of a thorough study of magnetotransport in ${\mathrm{Cd}}_{1-x}{\mathrm{Hg}}_{x}\mathrm{Te}/{\mathrm{Hg}}_{1-t}{\mathrm{Cd}}_{t}\mathrm{Te}/{\mathrm{Cd}}_{1-y}{\mathrm{Hg}}_{y}\mathrm{Te}$ heterosystems with both an inverse band structure and a normal one, where the QWs are formed by different variations in the Cd (Hg) content. The original data demonstrating pronounced beatings of Shubnikov-de-Haas oscillations (SdH) and their scrupulous analysis are provided. The giant Rashba spin splittings and the dependence of total spin-splitting on the magnetic field are obtained through the Fourier analysis of oscillations and by analyzing the positions of the beating nodes in a magnetic field, respectively.

## 2. Materials and Methods

Our ${\mathrm{Cd}}_{1-x}{\mathrm{Hg}}_{x}\mathrm{Te}/{\mathrm{Hg}}_{1-t}{\mathrm{Cd}}_{t}\mathrm{Te}/{\mathrm{Cd}}_{1-y}{\mathrm{Hg}}_{y}\mathrm{Te}$ QWs are grown by molecular beam epitaxy on GaAs (013) substrates [25,26,27,28,29]. The QWs are asymmetrically modulation doped with In in bottom barriers (on the substrate side), with a concentration *n*_imp_ = 1.8 × 10^18^ cm^−3^. We measure two structures, which differ by various widths of the forming layers and by Cd(Hg) content. The first QW is ${\mathrm{Cd}}_{0.89}{\mathrm{Hg}}_{0.11}\mathrm{Te}/{\mathrm{Hg}}_{0.85}{\mathrm{Cd}}_{0.15}\mathrm{Te}/{\mathrm{Cd}}_{0.85}{\mathrm{Hg}}_{0.15}\mathrm{Te}$ (0.89/0.15/0.85) (Sample N), the second QW—${\mathrm{Cd}}_{0.6}{\mathrm{Hg}}_{0.4}\mathrm{Te}/{\mathrm{Hg}}_{0.95}{\mathrm{Cd}}_{0.05}\mathrm{Te}/{\mathrm{Cd}}_{0.53}{\mathrm{Hg}}_{0.47}\mathrm{Te}$ (0.6/0.05/0.53) (Sample I). The ${\mathrm{Cd}}_{1-x}{\mathrm{Hg}}_{x}\mathrm{Te}$ bottom barrier is composed of a spacer, a doped layer and one more barrier layer. Then, a buffering CdTe and ZnTe layers follow. Above QWs, there are a ${\mathrm{Cd}}_{1-y}{\mathrm{Hg}}_{y}\mathrm{Te}$ barrier layer and a CdTe cap layer. The parameters of the sample structures are summarized in Table 1. Standard Hall bars are fabricated by wet chemical etching. Ohmic indium contacts are made by thermal soldering. Magnetotransport measurements are carried out in a He4 cryostat using DC techniques with a current of 1 μA in magnetic field *B* up to 9 T in Quantum Design measuring system and up to 12 T in Oxfords Instruments setup at temperature 1.8 K.

**Figure 1:**
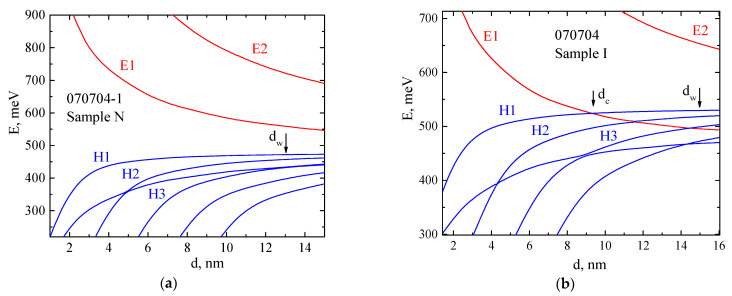
Energies of size-quantized subbands at *k* = 0 as a function of the ${\mathrm{Hg}}_{1-t}{\mathrm{Cd}}_{t}\mathrm{Te}$ quantum well width for *t* = 0.15 (Sample N) (**a**) and *t* = 0.05 (Sample I) (**b**).

${\mathrm{Cd}}_{1-y}{\mathrm{Hg}}_{y}\mathrm{Te}$${\mathrm{Hg}}_{1-t}{\mathrm{Cd}}_{t}\mathrm{Te}$${\mathrm{Cd}}_{1-x}{\mathrm{Hg}}_{x}\mathrm{Te}$${\mathrm{Cd}}_{1-x}{\mathrm{Hg}}_{x}\mathrm{Te}$ The calculations of the band structure for heterostructures close to those studied here both in composition and in the width of quantum wells were performed in [25] within the framework of the 8-band Kane model taking into account BIA and IIA. Structural asymmetry, due primarily to asymmetric doping, was taken into account in the Hartree approximation. The calculation showed the inverted type of band structure in ${\mathrm{Hg}}_{0.95}{\mathrm{Cd}}_{0.05}\mathrm{Te}$ QW, the lower subband in the conduction band is formed mainly by the states of heavy holes Γ8, in contrast to ${\mathrm{Hg}}_{0.85}{\mathrm{Cd}}_{0.15}\mathrm{Te}$ QW, which has a normal band order and the lower subband in the conduction band E1 of it is formed mainly by the electron-like states Γ6 (see Figure 1 and insets on it in [25]).

The calculation [25] predicts a significant Rashba spin splitting in the lower subband of the conduction band. Taking into account the BIA and IIA effects gives an additional correction to this value, and the contributions of these effects are significantly different in subbands of different nature. In the H1 band, the spin splitting increases by 1–2 meV, which is a small value vs the Rashba splitting background, but in the E2 subband these effects give a larger splitting of 2–3 meV.

Within the framework of the same calculation procedure, as in Reference [25], we calculated the dependence of the energies of size-quantized levels on the QW width in our structures (Figure 1). In Sample I (Figure 1b), the band inversion point is at a width of *d_c_* = 8.5 nm, which is much larger than the critical width for a classical quantum well Hg_0.3_Cd_0.7_Te/HgTe/Hg_0.3_Cd_0.7_Te (0.7/0/0.7) [22,30]. For Sample N, with a larger cadmium contents both in the QW and in the barriers, this point is hardly reached (Figure 1a).

## 3. Results

**Figure 2:**
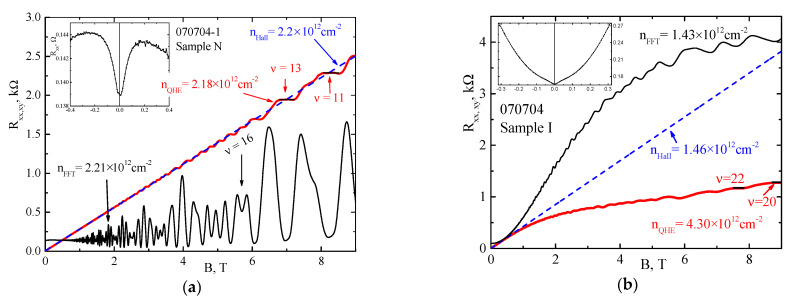
The dependencies of longitudinal, *R_xx_*, (black line) and Hall, *R_xy_*, (red bold line) resistivities on the magnetic field *B* at *T* = 1.8 K for Sample N (**a**) and Sample I (**b**). The QHE plateaus in *R_xy_* are marked with the corresponding filling factors *ν* = 11, 13 for Sample N and *ν* = 22, 20 for Sample I. Insets in (**a**,**b**) show the effect of weak antilocalization in both samples.

We analyze the components of the magnetoresistance tensor *R_xx_* and *R_xy_* measured in magnetic fields *B* up to 9 T at a fixed temperature *T* = 1.8 K. Both samples demonstrate a rich picture of SdH oscillations in magnetic fields from ~0.5 T to maximum fields of 9 T. However, the difference in the conductivity of the samples is also obvious. In Sample N, SdH oscillations are observed on the *R_xx_*(*B*) dependence with a weak, almost linear, monotonic background (Figure 2a), while Sample I demonstrates a pronounced parabolic dependence of the longitudinal resistance in a magnetic field range up to 2 T, followed by a tendency towards saturation at *B* > 6 T (Figure 2b). The dependences of the Hall resistance *R_xy_* on the magnetic field also significantly differ for the two samples. We reach the quantum Hall effect (QHE) plateaus with numbers, *i* = *h*/(*e*^2^*R_xy_*), *i* = 11 (Sample N) and *i* = 20 (Sample I). Electron density, calculated from the position of the mentioned plateaus, are as follow: *n_QHE_* = 2.18 × 10^16^ m^−2^ (Sample N) and *n_QHE_* = 4.30 × 10^16^ m^−2^ (Sample I). For Sample N, we see the traditional QHE picture (red bold line in Figure 2a), in which the plateaus and the transition regions between them in the *R_xy_*(*B*) dependence are centered around an inclined straight line, extrapolated from the classical Hall magnetoresistance in the low field region (blue dashed straight line in Figure 2a). *R_xy_*(*B*) corresponds to the density of a two-dimensional electron gas *n_Hall_* = 1/(*R_H_* × *e*) = 2.20 × 10^16^ m^−2^ (*R_H_* = *R_xy_*(*B*)/*B* is the Hall constant), which is in excellent agreement with the QHE data. In Sample I, the deviation of the *R_xy_*(*B*) dependence (red bold curve in Figure 2b) at *B* > 1 T from the classical Hall magnetoresistance (blue dashed straight line in Figure 2b) corresponds to the behavior of the Hall resistance for two types of electrons with different mobilities, as just seen for the *R_xx_* behavior (see discussion in Appendix A). The electron density is *n_Hall_* = 1/(*R_H_*×*e*) = 1.46 × 10^16^ m^−2^, which is almost three times less than the *n_QHE_* obtained from the QHE data.

A well-pronounced characteristic positive magnetoresistance is observed at very low perpendicular magnetic fields for both Sample N at *B* < 0.2 T and for Sample I at *B* << 0.1 T (see insets on Figure 2a,b, respectively), which is attributed to the effects of weak antilocalization (WAL). The WAL effect originates from the breaking of the spin coherence in magnetic fields in the presence of zero-field spin-splitting, Δ*_R_*, arising from the spin-orbit interaction. The discussion on WAL will be presented elsewhere.

**Figure 3:**
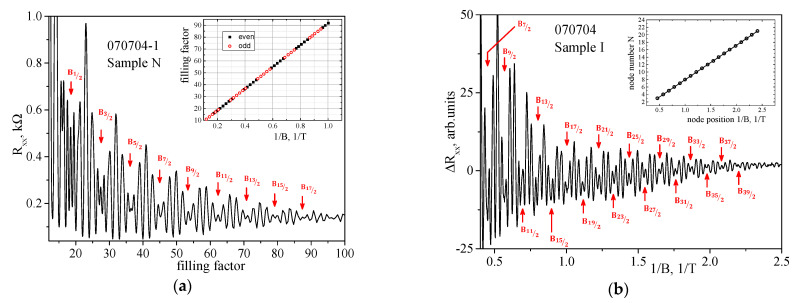
The oscillating part of *R_xx_* curves (shown on Figure 2) vs filling factor for Sample N (**a**) and 1/*B* for Sample I (**b**). Node positions are denoted by the arrows. The numbering of nodes is indicated near the arrows. Insets: (**a**) Dependence of the oscillation minima positions in 1/B on the filling factor *ν* for Sample N. Filled squares denote even numbers; open circles denote odd ones. (**b**) Dependence of oscillation node positions in 1/B on its numbers N for Sample I.

Figure 3a for Sample N shows the dependence of *R_xx_* on the filling factor ν, ν = *n_s_/n_B_*~1/*B*, where *n_s_* and *n_B_* = *eB*/*h* are the electron density and the number of states at the Landau level per unit area, respectively. The oscillations are periodic in the reciprocal magnetic field and have pronounced beating nodes. The positions of the *R_xx_* (*B*) oscillation minima correspond to the integer values of the filling factor. To analyze the phases of the oscillations, we plot the dependence of the values of the reciprocal magnetic fields, 1/*B*_min_, corresponding to the oscillation minima, on the filling factor ν (inset in Figure 3a). It can be seen that the position of the oscillation beating node corresponds to a change in the parity of the filling factors (even and odd minima are indicated by different symbols, inset in Figure 3a). This change in the parity corresponds to a change by π of the oscillation phase, i.e., to a beating node. In quasi-classical terms, the presence of beats in the SdH oscillations indicates the existence of two close Fermi surfaces, which correspond to close values of oscillation frequencies. In the case of 2D electron and hole systems with one filled size-quantized subband, the occurrence of two close Fermi surfaces (contours in the 2D case) is usually attributed to the removal of spin degeneracy in a zero magnetic field and the appearance of the associated difference in the densities of charge carrier states on different branches of the dispersion law. Areas of mixing for different symbols in the inset to Figure 3a at ν < 40 respond to the appearance of Zeeman splitting in the spectrum of Landau levels with an increase in the magnetic field |ℏω*_c_*_−_*gμ_B_B*| > Γ, where Γ is the broadening of the Landau level, ω*_c_* = *eB/m_c_*, *m_c_* is the cyclotron effective mass, the effective *g-*factor, *μ_B_* = *e*ℏ*⁄*(2*m*_0_) is the Bohr magneton.

## 4. Discussion

### 4.1. Fourier Analysis of Oscillations

**Figure 4:**
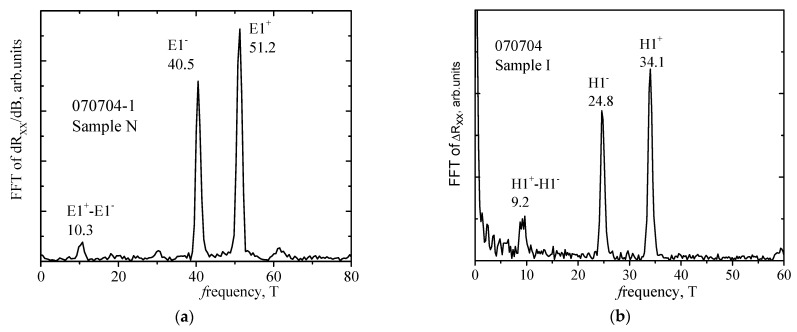
FFT spectra of SdH oscillations of Sample N (**a**) and Sample I (**b**). The Fourier peaks are labeled as E1^+^ and E1^−^ for Sample N and H1^+^ and H1^−^ for Sample I according to corresponding spin-split subbands. Differential peaks are denoted as well.

One of the main methods of the frequency analysis of oscillations is the Fast Fourier Transform (FFT) of *R_xx_* data to determine the characteristics of oscillations, such as frequency, phase and amplitude. For a detailed analysis of the SdH oscillations, we subtract the monotonous part of the *R_xx_*(*B*) dependence. This can be carried out in two ways: by subtracting the polynomial, the degree of which is selected as the best centering of the oscillations along the x axis (see Figure 3b) or by numerical differentiation of the data. Both ways of processing were used and the results were compared, so the most pictorial presentation of the data is given in the paper. The results of the Fourier analysis of the *R_xx_*(1/*B*) data for Sample N (Figure 3a) are shown in Figure 4a. It can be seen that only two close frequencies *F* are resolved, which correspond to the spin-orbit splitting of the E1 subband. In addition to the main peaks, designated E1^+^ and E1^−^, there are other peaks that correspond to the combinations of frequencies from the main peaks. The sum of the densities obtained from the frequencies of peaks E1^+^ and E1^−^ gives *n_FFT_* = 2.21 × 10^16^ m^−2^, (*n_FFT_* = $\frac{e}{h}$*F* = $\frac{e}{h}$(*F*^+^ + *F*^−^)), which ideally corresponds to the data for this sample that were obtained from other regions of magnetic fields. The parameters of the samples under study are summarized in Table 2.

Figure 3b for Sample I shows the dependence of the oscillating part Δ*R_xx_* = *R_xx_* − *R_mon_* with a residue of the monotonic part approximated by a polynomial, *R_mon_* = $\underset{m}{\sum }1/B^{m}$, on the reciprocal magnetic field 1/*B*. In Sample I, it can be seen that the oscillations are periodic in the reciprocal magnetic field and have pronounced beating nodes. However, for this sample, it was not possible to establish the correspondence of the positions of the oscillation minima to the integer values of the filling factor ν, since electrons with lower mobility participate in the conductivity (see discussion in Appendix A). The results of the Fourier analysis of the data Δ*R_xx_*(1/*B*) for Sample I (Figure 3b) are shown in Figure 4b. Again, only two close frequencies are resolved, which correspond to the spin-orbit splitting of the *H*1 subband. The sum of the densities obtained from the frequencies of peaks *H*1^+^ and *H*1^−^ gives *n_FFT_* = 1.43 × 10^16^ m^−2^, which ideally corresponds to the data obtained from the low magnetic fields n_Hall_ (see Table 2). In addition to the main peaks, designated *H*1^+^ and *H*1^−^, there are other peaks that correspond to combined frequencies. Thus, the low-frequency peak at *f*_3_ = 9.3 T is a difference peak from the main peaks *H*1^+^–*H*1^−^ = (34.1–24.8) T.

Winkler [31] showed that spin-splitting energy should be proportional to *k*_||_^3^ for a *p-*type state (heavy holes) Γ8 with *J_z_* = ±3/2, but for an electron-like state of s-type Γ6, *J_z_* = ±1/2, and a light hole state Γ8 with *J_z_* = ±1/2, spin-splitting should be a linear function of wave number *k*_||_. Then, for *p-*type states with *J_z_* = ±3/2 for the parameters of the Rashba spin-orbit splitting, we have:

(1) εΓ8SO=±〈βEz〉k∥3, βEz=ℏ22m*X(2−X)4πn, X=2(2+1−a2)a2+3, a=Δnn, Δn=n+−n−, n=n++n−, ΔRΓ8=2〈βEz〉kF3 

For the *s*-type states with *J_z_* = ±1/2 the parameters of the Rashba spin-orbit splitting are given:

(2) $$\varepsilon_{\Gamma_{6}}^{SO}=\pm \langle \alpha E_{z}\rangle k_{∥},\;\alpha E_{z}=\frac{\hbar^{2}}{m^{*}}\sqrt{\frac{\pi }{2}}\frac{\Delta n}{\sqrt{n-\Delta n}},\;\Delta_{R}^{\Gamma_{6}}=2\langle \alpha E_{z}\rangle k_{F},$$

where $\varepsilon_{\Gamma_{6,8}}^{SO}$ are the energies of spin-orbit splitting states with different symmetries, *n* and Δ*n* are the sum and difference of electron densities in the aforementioned states, *α*, *β* are the spin-orbit interaction constants, and *E_z_* is the effective electric field in the *z* direction.

Based on the difference in carrier densities in spin-split subbands, Δ*n*, and knowing the effective mass of charge carriers, it is possible to determine the parameters of the Rashba spin-orbit interaction—the spin-orbit interaction constant, *αE_z_* and *βE_z_*, and the spin-orbit splitting energy at zero magnetic field, Δ*_R_*. The effective electron masses in the spin-split subbands of the studied structures were determined by the cyclotron resonance: Sample I, m_c_^−^ = 0.0376 *m*_0_, *m_c_*^+^ = 0.0417 *m*_0_ [25], and Sample N, *m_c_*^−^ = 0.0459 *m*_0_, *m_c_*^+^ = 0.0498 *m*_0_ [25]. To estimate the required parameters of the Rashba spin-orbit splitting in the samples under study, the average value of the effective mass m^*^ ≈ 0.039 *m*_0_ (Sample I) и and m^*^ ≈ 0.048 *m*_0_ (Sample N) can be used. Using the expression (1) for Sample I, we found *βE_z_* = 4.97 × 10^−19^ meV·cm^3^, Δ*_R_*^Γ^_8_ = 26.6 meV, and using the expression (2) for the Sample N, we obtained *αE_z_* = 37 × 10^−12^ eV·m, Δ*_R_*^Γ^_6_ = 27.4 meV (Table 2).

### 4.2. Analysis of the Beating Node Positions of SdH Oscillations in Magnetic Field

Let us use another way to determine the Rashba spin-splitting. In the quantum mechanical consideration, the occurrence of beatings of SdH oscillations is due to the presence of two types of energy splits in the spectrum (cyclotron and Zeeman) and the dependence of their ratio on the magnetic field. In our case, spin-orbit splitting in the zero field is also added. The specific SdH oscillation beating pattern observed in the experiment (Figure 3) is associated with the overlap of oscillations that are close in frequency from two spin-split subbands. Modulation of the amplitude of the SdH oscillations in this case is determined by cos(πδ/ℏ*ω_c_*); the beating nodes correspond to the points where cos amplitude is zero, which is the case when [32]

(3) $$\frac{\delta }{\hbar \omega_{c}}\;=\;N+\frac{1}{2},\;N=0,\;1,\;2,\ldots ,$$

where *δ* is the full spin-splitting,

(4) $$\delta \left(B\right)=\Delta_{R}+\delta_{1}\hbar \omega_{c}+\delta_{2}{\left(\hbar \omega_{c}\right)}^{2}+\ldots .$$

The term quadratic in the field and higher-order terms become important in strong magnetic fields; in other cases, it is possible to restrict ourselves to the first two terms. The last beating node of SdH oscillations with increasing magnetic field is at *δ*/*ℏω_c_* =1/2. Thus, the field dependence of the total spin-splitting *δ*(*B*) can be obtained from the position in the magnetic field of the SdH oscillation beating nodes.

There are two ways to put numbers *N* for the beating nodes. Formula (3) leads to a simple recurrence relation for the position in the magnetic field of the beating nodes [33]:

(5) $$B_{N+\frac{1}{2}}/B_{N+\frac{3}{2}}=\left(N+3/2\right)/\left(N+1/2\right)=\left(2N+3\right)/\left(2N+1\right).$$

Thus, the positions in the magnetic fields in which the neighboring nodes are located refer to each other as the nearest odd numbers. If the experimental conditions make it possible to observe the beating nodes corresponding to small *N*, then using this procedure allows to the nodes to be unambiguously numerated.

**Figure 5:**
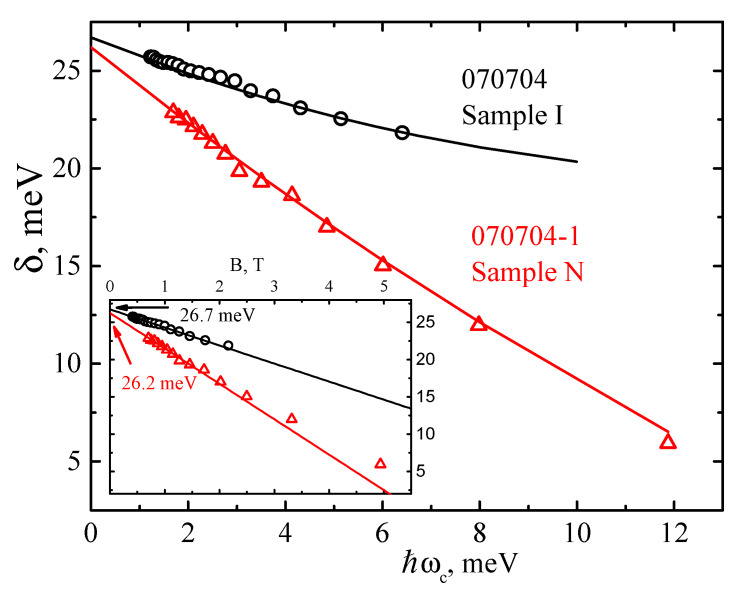
Total experimental spin-splitting energies *δ* for Sample N (triangles) and Sample I (circles) as a function of $\hbar \omega_{c}$. Lines are the fitting according to Equation (10). Inset shows the low field limit, $\hbar \omega_{c}\ll \frac{\Delta_{R}}{1-\frac{gm^{*}}{2m_{0}}}$, of Equation (10). The Rashba spin-orbit splitting energies at *B* = 0, Δ*_R_*, obtained by low field fitting are indicated by arrows.

Figure 3 shows the numbering of the beating nodes of the SdH oscillations in the studied samples using the recurrence relation (5). In Sample N, there are 14 nodes and the node with *δ*/*ℏω_c_* = 1/2 is visible. Sample I contains 19 nodes, with node *δ*/*ℏω_c_* = 7/2 corresponding to the largest magnetic fields. Thus, by knowing the numbers of the SdH oscillation beating nodes, one can obtain the field dependence of the total spin-splitting *δ*(*B*) (Figure 5; see discussion below).

The second method of node numbering is based on plotting the position of the beating nodes in a reciprocal magnetic field, 1/*B*, vs the node number, *N* (inset in Figure 3b). Expression (3) is rewritten in the following form

(6) $$\delta_{N}\left(B\right)=\left(N+\frac{1}{2}\right)\hbar \omega_{c}=\frac{\hbar e}{m^{*}}B_{N}\left(N+\frac{1}{2}\right)$$

and, limiting only to the linear term in expression (4), we obtain

(7) $$N+\frac{1}{2}=\frac{m^{*}}{\hbar e}\Delta_{R}\frac{1}{B_{i_{N}}}+\delta_{1}$$

which describes the plot in the inset to Figure 3b. The slope of the resulting straight line is determined by Δ*_R_*; therefore, the nodes can be numbered in an arbitrary way. The slope of the straight line does not depend on this. Correctly numbered nodes give the proper intercept on the y-axis. If the numbers of the SdH oscillation beating nodes are chosen correctly, then the free term of expression (7) makes it possible to determine the coefficient at the *ℏω_c_*.

The formula for the position of the beating nodes, which is analog of Equations (6)–(7) and allows for the parameters of the Rashba splitted band spectrum to be determined, was obtained in References [34,35].

The Δ*_R_* values obtained from the *N*(1/*B_N_*) plot (Figure 3) are as follows: 30.3 meV (Sample N) and 22.2 meV (Sample I), which is in good agreement with the values obtained using the Fourier analysis (Table 2).

### 4.3. Disscusion of Rashba Parameter Values

Let us analyze the results that were obtained for the Rashba spin-orbit splitting. It is worth noting that the constants of the Rashba SO interaction, *α* and *β*, are determined by the band structure parameters of the bulk semiconductor, namely [31,36,37]:

(*α*, *β*) ≈ Δ/|*ε_g_*|. (8)

Here, Δ = *E*_Γ8_ − *E*_Γ7_ is the spin-orbit splitting of the Γ8 and Γ7 bands in the crystal, *ε_g_* = *E*_Γ6_ − *E*_Γ__8_ is the energy gap between the Γ6 and Γ8 bands in crystals with narrow-gap (*ε_g_* > 0) and gapless (*ε_g_* < 0) semiconductors. According to (8), a strong Rashba spin-orbit coupling appears in 2D structures based on narrow-gap (CdHgTe) and gapless (HgTe) semiconductors, due to their small |*ε_g_*|.

It should be noted that the Δ*_R_* values obtained in the present study for structures with different types of band structure are almost the same. This is apparently due to the large fraction of *p-*type wave functions in the *E*1 subband of Sample N (see the inset in Figure 1b [25]). For the states of the *H*1 subband of the Sample I, the contribution of the *p-*type states to the eight-component wave function reaches 50%; for the *E*1 subband of Sample N, this fraction is less, but close to 40%. Calculations give the spin-splittings at the Fermi level Δ*_R_*^calc^ = 21 (Sample I) and 17 (Sample N) meV [25], which is significantly less than the experimental results of this work.

In the pioneering work [11], the Rashba spin-splitting in HgTe SQW’s with an inverted band structure was investigated on the gate-controlled Hall devices. The QW’s were modulation doped symmetrically, on both sides of the HgTe QW, in one sample with a well width of 21 nm, and asymmetrically, only on the substrate side of the HgTe QW, in the other, with a well width of 12 nm, using CdI_2_ as a doping material. For the symmetrically doped case of (0.49–1.3) × 10^12^ cm^−2^ gate-control densities, the spin-orbit constant values changed from 8 × 10^−19^ meV·cm^−3^ to zero and then increased to 2 × 10^−19^ meV·cm^−3^. In the present work, with an asymmetric doped QW (Table 2), the density in the spin-splitted subbands, *n_s_* = 1.43 × 10^16^ m^−2^, exceeds the upper limit for density in Reference [11] and, correspondingly, the larger *βE_z_* parameter value was obtained.

The largest value of the Rashba spin-orbit splitting known in structures with rectangular HgTe QWs to date is 30 meV, observed in the 12.5-nm-wide HgTe/Hg_0.3_Cd_0.7_Te (001) QW with a density of 2.7 × 10^12^ cm^−2^ doped asymmetrically on the top barrier of the QW, using CdI_2_ as a doping material [12]. It was underlined that this large Δ*_R_* in HgTe QWs with an inverted band structure is caused by its narrow gap, the large spin-orbit gap between the bulk valence bands Γ8 and Γ7, and the heavy-hole character of the first conduction subband. The value of Reference [12] is quite close to the result of our work for Sample I with inverted band structures; thus, the same reasons for their large Rashba spin-splitting should be valid here.

The Rashba spin-splitting, as a function of carrier density, was investigated in a high-mobility 2DEG formed in *p-*type Hg_0.77_Cd_0.23_Te inversion layers with a normal band structure using WAL analysis [24]. The values of Δ*_R_* and *α* were 2–10 meV and 8–24 × 10^−12^ eV·m in the density range from 3 × 10^11^ cm^−2^ to 6 × 10^11^ cm^−2^. It was found that both Δ*_R_* and *α*, obtained from the experiment, were much smaller than the values predicted by the linear Rashba model. This discrepancy was explained by the nonlinear Rashba effect, which was caused by the weakening of interband coupling between the valence and conduction bands with increasing *k*. In our study, both Δ*_R_* and *α* were far larger than the known values [24], and this was associated with the different carrier densities in the compared samples.

The results for Δ*_R_* in HgTe-based QW’s are summarized in Figure 4 of Reference [15]. One can see that the Δ*_R_* values for structures with a normal band structure were within 10 meV, while the QW’s with inverted band structure had far larger values, of up to ~34 meV, which were obtained in two-dimensional electron gas confined in inversion layers on Hg_1−*x*_Cd*_x_*Te with an inverted band structure (*x* = 0.10 − 0.09), with electron densities of 1.32 × 10^12^ cm^−2^ and 1.55 × 10^12^ cm^−2^ in two samples [15].

### 4.4. Magnetic Field Dependence of the Total Spin Splitting

In magnetic field *B*, the energy spectrum for the *n*-th Landau level may be presented, considering both the Zeeman spin splitting, $\Delta_{Z}=g\mu B$, and the spin-orbit splitting of Rashba, Δ*_R_*, as [38]:

(9) $$\begin{array}{l} E_{0}=\frac{1}{2}\hbar \omega_{c}\;\mathrm{for}\;n=0,\\ E_{n}^{\pm }=\hbar \omega_{c}\left[n\pm \frac{1}{2}\sqrt{{\left(1-\frac{gm^{*}}{2m_{0}}\right)}^{2}+n\frac{\Delta_{R}^{2}}{E_{F}\hbar \omega_{c}}}\right].\; \end{array}$$

Then, the total spin splitting, δ(*B*), is determined by the expression [38]:

(10) $$\delta \left(B\right)={\left[{\left(\hbar \omega_{c}-g\mu_{B}B\right)}^{2}+\Delta_{R}^{2}\right]}^{1/2}-\hbar \omega_{c}≅\{\begin{array}{c} \Delta_{R}-\hbar \omega_{c},\;\mathrm{if}\;\hbar \omega_{c}\ll \;\frac{\Delta_{R}}{1-\frac{gm^{*}}{2m_{0}}}\;\\ \frac{gm^{*}}{2m_{0}}\hbar \omega_{c},\;\mathrm{if}\;\hbar \omega_{c}\gg \;\frac{\Delta_{R}}{1-\frac{gm^{*}}{2m_{0}}}\; \end{array}$$

The result of Equation (10) can be understood in the following way [38]. In high magnetic fields, the spin-orbit-coupled states are strongly separated in energy, which significantly reduces the coupling effect, and the spin-splitting *δ* (Equation (10)) approaches the Zeeman splitting. In low magnetic fields, the spin-splitting *δ* (Equation (10)) linearly decreases with $\hbar \omega_{c}$; the slope of this linear reduction is determined by the effective mass of charge carriers. In high magnetic fields, Zeeman splitting predominates, and this limit can be used to estimate the *g*-factor.

The theoretical model [38] was developed for the two spin-splitting mechanisms of Dresselhaus (BIA) and Rashba, and it was shown that only accounting for BIA or assuming that the BIA term has a comparable strength to the Rashba effect significantly deteriorated the agreement with the perpendicular-field experimental data. The key role of Rashba term was revealed, and the analytical expression Equation (10) was obtained.

The low field limit of Equation (10) is examined in the inset of Figure 5. For Sample N with a normal band structure, a deviation from linear dependence of δ(B) was observed at *B* > 1.5 T. For Sample I, the low field limit is well-described by the calculated values of *δ_N_* in approximately the whole range of the magnetic field. The values of $\Delta_{R}$, obtained by approximating the dependence *δ*(*B*) to *B* → 0, coincide well with the values obtained from the Fourier analysis and the *N*(1/*B*) plot (see Table 2). In Figure 5, the spin-splitting, as a function of $\hbar \omega_{c}$, is presented for the two samples under study. It can be seen that a good description by theoretical Equation (10), with effective masses and *g*-factors as a fitting parameters, is observed for both QWs. The following parameters were obtained for the theoretical curves (solid lines in Figure 5): $m_{corr}^{*}=0.039m_{0}$, |g|=35 (Sample I) and $m_{corr}^{*}=0.024m_{0}$, |g|=30 (Sample N). An adequate description of the experimental data by the theoretical dependence (10) indicates the predominance of the Rashba contribution to the spin-orbit splitting at *B* = 0 than the contribution of Dresselhaus.

The importance of the experimental determination of the effective mass and *g*-factor in systems with a sophisticated dispersion law, to which the structures based on HgTe belong, is beyond doubt. The previously obtained experimental values of the *g-*factor and effective mass for HgTe quantum wells are contradictory and depend on the width of the quantum well and the electron density [39,40,41,42,43,44,45,46]. It was previously noted [39,40,41,42] that there is a problem of accordance between theoretical and experimental estimates of the effective mass in such QWs. If we systematize the currently available numerous experimental estimates of the effective mass in QWs based on HgTe, it is shown that with an increase in the density of charge carriers from ~2 × 10^15^ m^−2^ to ~1 × 10^16^ m^−2^ $m^{*}/m_{0}$ increases from ~0.020 to (0.026–0.034) (see, for example, [42,43,44,46]). In the structures with a normal spectrum, the experimental values of the effective mass are close those calculated by the ***kP*** method over the whole density range; with the increasing QW width, in an inverted spectrum regime, the experimental values of effective mass become noticeably smaller than the calculated ones [42]. The values of $m^{*}/m_{0}$ and g-factors obtained by fitting *δ*(*B*) dependence with Equation (10) are in good accordance with the previously obtained results for HgTe QWs.

## 5. Conclusions

Studies were carried out on quantum magnetotransport in heterostructures based on CdHgTe solid solutions, with a varying Cd (Hg) content in both the quantum well and the barriers. It is possible to realize systems with both normal and inverted energy spectra by compositional variations in quantum wells with a similar width.

We used several methods to estimate the Rashba spin-splitting, and then compared the results. Estimations for both the normal and inverted band spectra were made: from the difference in the carrier concentration in the spin-split subbands found by the Fourier analysis of SdH oscillations and from the analysis of the beating node positions of SdH oscillations in both low magnetic fields and the wider range of magnetic fields. Parameter estimates using different methods were in good agreement with each other.

Due to the high quality of the samples and the high concentration of electrons (*n* > 1.4 × 10^12^ cm^−2^), a remarkably rich SdH oscillation pattern is observed in our structures: high-resolution oscillations with well-defined beating nodes for numbers from *N* = 0 ($\delta /\hbar \omega_{C}=1/2$) up to *N* = 14 and *N* = 19 in QWs with the normal and inverted energy spectra, respectively.

This allowed to describe the experimental data for the total spin-splitting, δ(*B*), in a wide range of magnetic fields using a theoretical expression, considering both Zeeman and Rashba effects with the effective mass values and the *g*-factor as the fitting parameters. The very fact of the adequate description of the data obtained using this theoretical dependence indicates the predominant Rashba contribution to the spin-orbit splitting at *B* = 0 in comparison with the contribution of Dresselhaus.

We note that the large Rashba splitting at *B* = 0 obtained in the present study for structures with different types of band structure is almost the same (Δ*_R_*~25÷27 meV), due to the significant fraction of *p-*type wave functions in both the *E*1 subband of the 070704-1 structure and the *H*1 subband of the 070704 structure.

## Figures and Tables

**Table 1:** Structural parameters of the samples under study.

Layer Order	070704-1 (Sample N)		070704 (Sample I)	
	Width, nm	Cd Content	Width, nm	Cd Content
CdTe cap	37	1	40	1
${\mathrm{Cd}}_{1-y}{\mathrm{Hg}}_{y}\mathrm{Te}$ barrier	32	0.85	31.5	0.53
${\mathrm{Hg}}_{1-t}{\mathrm{Cd}}_{t}\mathrm{Te}$ QW	13	0.15	15	0.05
${\mathrm{Cd}}_{1-x}{\mathrm{Hg}}_{x}\mathrm{Te}$ spacer	9.5	0.89	11.5	0.6
In doped layer *n*_imp_ = 1.8 × 10^18^ cm^−3^	10	0.89	13.5	0.6
${\mathrm{Cd}}_{1-x}{\mathrm{Hg}}_{x}\mathrm{Te}$ barrier	5	0.89	5.5	0.6
CdTe buffer	6000	1	6000	1
ZnTe buffer	30		30	
GaAs (013) substrate	4 × 10^5^		4 × 10^5^	

**Table 2:** Electron gas and Rashba spin-orbit parameters of the samples under study.

Parameter (Definition Method)		070704-1 Sample N	070704 Sample I
*n*_QHE_ (QHE)		2.18 × 10^16^ m^−2^	4.30 × 10^16^ m^−2^
*n*_Hall_ (*n*_Hall_ = 1/(*R_H_*·*e*))		2.20 × 10^16^ m^−2^	1.46 × 10^16^ m^−2^
*n*_FFT_ (FFT)		2.21 × 10^16^ m^−2^	1.43 × 10^16^ m^−2^
*n* (two types of electrons)	*n*_1_	—	1.40 × 10^16^ m^−2^
	*n*_2_		3.20 × 10^16^ m^−2^
	*n* = *n*_1_ + *n*_2_		4.60 × 10^16^ m^−2^
*μ*		2.0 m/(V·s)	
*μ* (two types of electrons)	*μ*_1_	—	24.5 m/(V·s)
	*μ*_2_		0.13 m/(V·s)
Δ*_R_* (FFT)		27.4 meV	26.6 meV
Δ*_R_* (*N*(1/*B_N_*) plot)		30.3 meV	22.2 meV
$\Delta_{R}\;(\mathrm{low}\;\mathrm{field}\;\mathrm{limit}\;\mathrm{of}\;\mathrm{Equation}\;(10)\;\hbar \omega_{c}\ll \frac{\Delta_{R}}{1-\frac{gm^{*}}{2m_{0}}}$)		26.2 meV	26.7 meV
Δ*_R_*^calc^ [25]		17 meV	21 meV
*α* (FFT)		$37\times 10^{-12}\;\mathrm{eV}\cdot m$	—
*β* (FFT)		—	$4.97\times 10^{-19}\mathrm{meV}\cdot {\mathrm{cm}}^{3}$
$m^{*}/m_{0}$ (Equation (10))		0.024	0.039
$m_{c}^{*}/m_{0}$ [25]		0.048	0.039
|g| (Equation (10))		30	35

**Figure A1:**
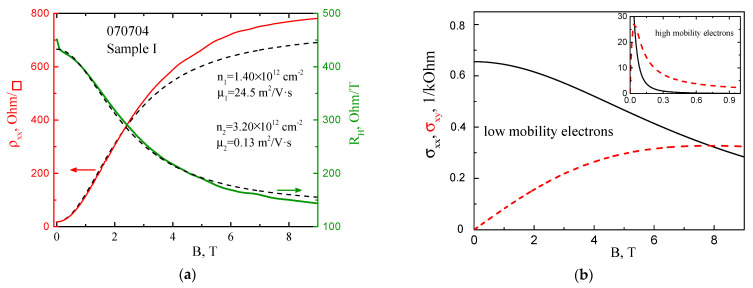
(**a**) Dependences of the monotonous part of *ρ*_xx_ and Hall constant, *R_H_*, on magnetic field *B* (solid lines) for Sample I and their fitting by Equation (A2) (dash lines). The resulting fitting procedure parameters are also displayed. (**b**) Dependences of *σ_xx_* (solid lines) and *σ_xy_* (dash lines) on magnetic field *B* for the low-mobility and high-mobility (inset) electrons, plotted with Equation (A1) using parameters from fitting procedure (Figure A1a).

## Data Availability

The data that support the findings of this study are available from the corresponding author upon reasonable request.

## Appendix A

As mentioned previously, two types of carriers, with significantly different mobilities, participated in the conduction for Sample I, as was concluded from the shape of the *ρ**_xx_*(*B*) and *ρ**_xy_*(*B*) curves, and from the difference in the electron densities determined from the range of classical magnetic fields, SdH oscillations and QHE regime. Let us discuss this fact in detail. When several types of charge carriers take part in the transport phenomena, each of them makes an additive contribution to the conductivity tensor:

(A1) $$R_{H}=\frac{\sigma_{xy}/B}{\sigma_{xx}^{2}+\sigma_{xy}^{2}},\;\rho_{xx}=\frac{\sigma_{xx}}{\sigma_{xx}^{2}+\sigma_{xy}^{2}}\;\sigma_{xx}=\sum_{k}\frac{e_{k}n_{k}\mu_{k}}{1+\mu_{k}^{2}B^{2}},\;\sigma_{xy}=\sum_{k}e_{k}n_{k}\mu_{k}\frac{\mu_{k}B}{1+\mu_{k}^{2}B^{2}}$$

where $R_{H}$—Hall constant, *k*—summation index over the number of types of charge carriers. Hence, the expressions for *R_H_*(*B*) and *ρ**_xx_*(*B*) follow, and are used in the analysis of positive magnetoresistance and the Hall effect for presence of two types of electrons [21]:

(A2) $$R_{H}=\frac{n_{1}\mu_{1}^{2}+n_{2}\mu_{2}^{2}+\mu_{1}^{2}\mu_{2}^{2}B^{2}(n_{1}+n_{2})}{e[{\left(n_{1}\mu_{1}+n_{2}\mu_{2}\right)}^{2}+\mu_{1}^{2}\mu_{2}^{2}B^{2}{\left(n_{1}+n_{2}\right)}^{2}},\;\rho_{xx}=\frac{n_{1}\mu_{1}+n_{2}\mu_{2}+\mu_{1}\mu_{2}B^{2}(n_{1}\mu_{1}+n_{2}\mu_{2})}{e[{\left(n_{1}\mu_{1}+n_{2}\mu_{2}\right)}^{2}+\mu_{1}^{2}\mu_{2}^{2}B^{2}{\left(n_{1}+n_{2}\right)}^{2}}$$

where *n*_1_, *n*_2_, *μ*_1_, *μ*_2_—densities and mobilities of electrons of the first and second types.

Dependences *ρ*_xx_(B) with a smoothed oscillating part and *R_H_*(*B*) for Sample I are shown in Figure A1a. The *R_H_*(*B*) curve has a characteristic form for the conduction of two types of electrons with significantly different mobilities, as evidenced by two regions of *R_H_*(*B*) flattening at *B* < 1 T and *B* > 5 T. A small beak on the *R_H_*(*B*) curve at *B* < 0.2 T corresponds to the difference in the mobility of electrons from the spin-orbitally split sub-bands of the *H*1 level. Let us describe the dependencies *ρ**_xx_*(*B*) and *R_H_*(*B*) by expressions (A2), with the density and mobility of electrons of the first and second types *n*_1_, *n*_2_, *μ*_1_, *μ*_2_ used as the fitting parameters. The index *k* = 1 denotes the parameters of electrons of the spin-orbit split level *H*1, *n*_1_ = $n_{+}+n_{-}$, *μ*_1_ = $\mu_{eff}$, where the value of effective mobility, $\mu_{eff}$, can be roughly estimated from the relation $\mu_{eff\;}\approx \;\frac{n_{+}}{n_{1}}\mu_{+}+\frac{n_{-}}{n_{1}}\mu_{-}.$ The index *k* = 2 denotes the density and mobility of low-mobility electrons.

The fitting results are shown in Figure A1a by the dashed lines. It can be seen that the fitting curves describe the experimental data quite well. The obtained parameters are *n*_1_ = 1.40 × 10^12^ m^−2^, *μ*_1_ = 24.5 m^2^/V × s; *n*_2_ = 3.20 × 10^12^ m^−2^, *μ*_2_ = 0.13 m^2^/V·s. First, it should be noted that *n*_1_ is in excellent agreement with the *n_Hall_* and the *n_FFT_* (Sample I). Second, the total density of electrons of the first and second types, obtained from the fit *n* = *n*_1_ + *n*_2_ = (1.40 + 3.20) × 10^12^ m^−2^ = 4.60 × 10^12^ m^−2^, is in good agreement with the data from the QHE *n_QHE_* = 4.30 × 10^12^ m^−2^. Third, we can observe a huge mobility ratio *μ*_1_/*μ*_2_ = 24.5/0.13~200, which indicates that we are dealing with conductivity not in the second size-quantized subband, but parallel conductivity in the doped layer in the barrier. This assumption is also supported by the fact that the quantum well in Sample I is rather shallow in terms of the ratio of Cd content in the barriers and in the well of 0.6/0.05/0.53 (see Table 1) as compared with the classical structure 0.7/0/0.7 and with Sample N 0.89/0.15/0.85. Let us make a simple estimate of the two-dimensional carrier density in the doped layer: *n*_imp_^2D^ = *n*_imp_ × *d*_imp_ = 1.8 × 10^18^ [cm^−3^] × 13.5 × 10^−7^ [cm] = 4.05 × 10^12^ [cm^−2^], *n*_imp_^2D^ − *n*_1_ = (4.05 − 1.40) × 10^12^ = 2.65 × 10^12^ [cm^−2^]. *n*_imp_^2D^ is in fairly good agreement with the estimate of the carrier density from the QHE *n*_QHE_, and *n*_imp_^2D^−*n*_1_ is close to the value of *n*_2_ estimated from the fit (Figure A1a), which also confirms our assumption of parallel conductivity via the doped layer in the barrier.

Figure A1b shows the dependences of *σ**_xx_* and *σ**_xy_* on the magnetic field separately for the first and second types of electron—low-mobility (Figure A1b) and high-mobility electrons (inset in Figure A1b). It can be seen that the point *σ**_xx_* = *σ**_xy_* (Figure A1b), where *ω_c_**τ* = *μ**B* = 1, separating the region of low classical and quantizing magnetic fields, is in a magnetic field of 7.8 T; therefore, the low-mobility electrons really not contribute to the SdH oscillation picture.
